# The Light of the Stars

**Published:** 1882-01

**Authors:** 


### THE LIGHT OF THE STARS.

For a number of years the special work carried on at the Harvard Observatory, under the direction of Professor Pickering, has been the measurement of the intensity of the light of the heavenly bodies. Some of the results presented at a recent meeting of the Society of Arts, at the Institute of Technology, Boston, indicate measurements almost incredibly fine. The light which falls upon the earth from the satellites of Mars, for example, is about equivalent to what a man's hand on which the sun shone at Washington would reflect to Boston. The labor of measuring the brightness of all the visible stars was begun two years ago. It has since gone on at the rate of about 40,000 a year, and will be completed next fall.

